# Photodynamic Therapy for Superficial Esophageal Cancer Using an Excimer Dye Laser

**DOI:** 10.1155/DTE.1.99

**Published:** 1994

**Authors:** Seishiro Mimura, Toru Otani, Shigeru Okuda

**Affiliations:** 1 Department of Gastroenterology The Center for Adult Diseases Osaka Osaka 537 Japan; 2 Department of Gastroenterology The Center for Adult Diseases Osaka 1–3 Nakamichi Higashinari-Ku Osaka 537 Japan

## Abstract

In order to improve the therapeutic effectiveness of photodynamic therapy with Photofrin II and laser light for superficial esophageal cancer, we employed an excimer dye laser instead of an argon dye laser. Eight superficial esophageal cancer lesions (7 cases) were treated. Of these 8 lesions, 6 were cured by initial treatment, while one lesion required another treatment. The final rate of cure was 88% (7/8).


*Diagnostic and Therapeutic Endoscopy*, Vol. 1, pp. 99–105
Reprints available directly from the publisher
Photocopying permitted by license only

© 1994 Harwood Academic Publishers GmbH
Printed in Malaysia

### Photodynamic Therapy for Superficial Esophageal Cancer Using an Excimer Dye Laser

SEISHIRO MIMURA, TORU OTANI and SHIGERU OKUDA

*Department of Gastroenterology, The Center for Adult Diseases, Osaka, Osaka 537, Japan*

*(Received December 21, 1993; in final form April 15, 1994)*

In order to improve the therapeutic effectiveness of photodynamic therapy with Photofrin II and laser light for superficial esophageal cancer, we employed an excimer dye laser instead of an argon dye laser. Eight superficial esophageal cancer lesions (7 cases) were treated. Of these 8 lesions, 6 were cured by initial treatment, while one lesion required another treatment. The final rate of cure was 88% (7/8).

**KEY WORDS:** endoscopic treatment, excimer dye laser (EDL), hematoporphyrin derivative (HpD), photodynamic therapy (PDT), superficial esophageal cancer

### INTRODUCTION

Photodynamic therapy (PDT) is a new method to treat malignant tumors using hematoporphyrin derivative (HpD) as the photosensitizer and a laser as the excitation light source. It also can be employed endoscopically (Dougherty *et al.*, 1979; Hayata *et al.*, 1982). This method kills malignant cells through a photochemical reaction, rather than through heat. Because HpD has a higher affinity for malignant tissue than for normal tissue, after intravenous injection it is concentrated more and retained longer in malignant tissue than in normal tissue (Lipson and Baldes, 1960). Stimulation by low-power laser light to irradiate the tumorous lesion and surrounding area causes release of singlet oxygen from HpD, and malignant tissue can be destroyed selectively (Okuda *et al.*, 1984; Weishaupt *et al.*, 1976). Tumor ischemic necrosis as a result of microvascular occlusion is also involved in the tumorcidal process (Star *et al.*, 1986).

From 1981 to 1990, we have treated seven superficial esophageal cancer lesions (six cases) by PDT with an argon dye laser (ADL, models 171-08 and 375-03, Spectra-Physics, Mountain View, Calif., US). The results showed that PDT is very effective for all morphologic superficial esophageal cancer except for the protruding type. However, analyzing results according to the depth of cancer invasion in these seven lesions, the rate of cure for submucosal carcinomas was only 33% (1/3), whereas that of mucosal carcinomas was 100% (4/4) as shown in Table 1 (Mimura, Ichii and Okuda, 1991). These data can be interpreted to indicate that the ADL laser beam could not penetrate and supply sufficient energy to activate HpD in the submucosal layer.

In 1990, therefore, we investigated an excimer dye laser (EDL, Hamamatsu Photonics, Hamamatsu, Japan) as an excitation light source for PDT, because its low-power pulsed beam was expected to be more efficient at exciting HpD than continuous wave lasers such as ADL and high frequency pulsed lasers such as copper vapor dye laser (CuVDL). In animal tumors it was proved that EDL is superior to ADL in teams of photodynamic action (Okunaka *et al.*, 1992).

In this article, we first present our clinical data on PDT for the treatment of superficial esophageal cancer and present a representative case. Other endoscopic methods are also discussed, including a comparison of EDL and other laser equipment.

### MATERIALS

Eight superficial esophageal cancer lesions (seven cases) were treated by PDT with EDL. Surgery was not indicated because of high risk factors or old age. There were six men and one woman, ranging in age from 52 to 74 (mean,

Address for correspondence: Seishiro Mimura, M. D., Department of Gastroenterology, The Center for Adult Diseases, Osaka 1-3 Nakamichi, Higashinari-Ku, Osaka 537, Japan.

**Table 1** Rates of cure obtained by PDT with the ADL in superficial esophageal cancers, classified by depth

| *Depth of invasion* | *Number of cases* | *Number of cured cases (%)* |
|---|---|---|
| Mucosa | 4 | 4 (100%) |
| Submucosa | 3 | 1 (33%) |
| Total | 7 | 5 (71%) |

67) years. In all cases a histologic diagnosis of squamous cell carcinoma was obtained by biopsy, and the depth of cancer invasion was assessed by endoscopic findings and endoscopic ultrasonography. The follow-up periods ranged from 12 to 44 months.

### METHODS

The PDT procedure includes three important points, that is, administration of photosensitizers, laser light irradiation, and pre- and postprocedural management (Table 2).

### Administration of Photosensitizer

Two mg/kg of PHE (freeze-dried Photofrin II) was injected intravenously. The solution was made by dissolving 75 mg/v of PHE with 30 mL of 5% glucose solution.

The photosensitizers used in our institute were hematoporphyrin derivative (HpD, Photofrin I) and dihematoporphyrin ether/ester (DHE, Photofrin II). HpD is a complex mixture derived from porphyrin, whereas DHE is a mixture of the dihematoporphyrin ether and dihematoporphyrin ester purified from HpD. For initial studies, HpD was provided from Dr. T. J. Dougherty of Roswell Park Memorial Institute, Buffalo, New York, through Dr. Y. Hayata of Tokyo Medical College, for a project on Cancer Research funded by the Ministry of Health and Welfare, Japan. In later studies, HpD was obtained from Photofrin Medical Inc., Cheektowaga, N. J., and from The Queen Elizabeth Hospital, Woodville, S. Aust. 5011, Australia, and DHE was obtained from Photofrin Medical Inc., Raritan, N. J. From 1989 to 1990, a phase II study of PDT was performed in Japan, and DHE (manufactured for Quadra Logic Technologies, Vancouver, Canada, by Ortho Diagnostic Systems, Inc., Raritan, N. J.) was provided by Lederle, Japan. From 1990 to 1992, a phase III study was performed and completed in Japan (Kato *et al.*, 1993), and at present PHE is provided by Lederle, Japan, for compassionate use.

**Table 2** PDT procedure

1. Intravenous injection of 3 mg/kg of HpD or 2 mg/kg of PHE.
2. Approximately 50 hours later, the entire lesion plus a 5-mm width of marginal mucosa is irradiated with a laser beam at 630 nm wavelength transmitted endoscopically. Standard dose: 90 J/cm$^2$ with ADL, 60 J/cm$^2$ with EDL.
3. Patients are protected from hypersensitivity to light and are treated for the subsequently formed ulcer.

### Laser Light Irradiation

About 50 hours after intravenous injection, the entire lesion plus a 5-mm wide perimeter of mucosa was irradiated with an EDL beam at 630 nm wavelength transmitted endoscopically. The laser beam was condensed and led to a 400-μm single quartz fiber (Obara Kogaku, Tokyo, Japan) with a divergence of 20° at the simply cut fiber tip without lens or diffuser. This quartz fiber was encased in a Teflon tube of 2.0 mm in outside diameter to be able to pass through the working channel of the conventional fiberscope, and air was pumped at a rate of 50 to 100 mL/min into the free space between the fiber and the tube to protect the tip of the quartz fiber from contamination.

### Excimer Dye Laser

The ultraviolet laser beam of 308 nm wavelength, which is delivered from the XeCl excimer laser, pumps the dye laser with Rhodamine 640, which then can induce to emit a secondary laser beam of 630 nm wavelength by adjusting the concentration of the dye to 0.4 mM. The EDL (model PDT EDL-1, Hamamatsu Photonics, Hamamatsu, Japan) has the following characteristics: wavelength, 630 nm; pulse energy, 8 mJ; peak power, 800 kW; pulse width, 10 nsec; frequency of repetition, 20, 30, or 40 Hz (Hirano *et al.*, 1990).

### Filters Used in PDT

In PDT, an electronic endoscope is not applicable, because excessive amounts of red light at 630 nm wavelength for irradiation prevent the CCD chip from forming an image. A green filter should be placed on the eyepiece of the fiberscope to protect the naked eye from being dazzled during photoradiation. We used an acetate filter (model SP-15, Fuji Filter, Tokyo, Japan) for observation and photography. It is a green filter for sensitivity tests of X-ray film, with a peak transmission of 63% at 530 nm wavelength. The transmissivity rate at 630 nm is 2%. We also inserted a green filter (model SP-19) in front of the CCD camera in a fiberscope-TV system (model OTV-F2, Olympus, Tokyo, Japan). It is one of the filters for separating three primaries with a peak transmissivity of 75% at 520 nm and 17% at 630 nm (Fuji Film, 1993).

### Energy Intensity of Irradiation

The irradiation was delivered at a total dose of more than 60 J/cm$^2$. For wider lesions, the irradiation spot of about one cm$^2$ was first set on the anal side of the lesion, and after delivering the scheduled dose of light there, the field of irradiation was shifted to the remaining part so as to irradiate the entire region as uniformly as possible. The effective irradiation of all parts of the lesion was confirmed by observing mucosal change under direct vision at a pause during the irradiation. Uniform irradiation was difficult, especially with large lesions, because of esophageal peristalsis and respiratory movement. However, assuming uniform irradiation, the total energy density (J/cm$^2$) of the irradiated area was calculated by the following formulas for the ADL or EDL, respectively (Mimura, Ichii and Okuda, 1993).

In the continuous wave laser:

$$\frac{\text{output(W)} \times \text{time(sec)}}{\text{total irradiated area (cm}^2\text{)}}$$

In the pulsed laser:

$$\frac{\text{pulse energy(J/pulse)} \times \text{frequency(pulse/sec)} \times \text{time(sec)}}{\text{total irradiated area (cm}^2\text{)}}$$

### Pre- and Post-therapeutic Care

Soon after injection of PHE, the patients were placed in a dark room not only to protect them from photosensitivity disease but also to prevent the photosensitizer from losing its biologic effect. After PDT, the patients were kept in bed without oral feeding and were given continuous intravenous glucose and saline with an H2-blocker and antibiotics for two days to allow healing of ulcers. During this period, the patients were kept in dim light, and their faces and hands were coated with antisunburn cream. These restrictions were gradually reduced, and the patients returned to normal conditions after 1 week (Mimura and Okuda, 1993).

### Evaluation of Response

The response was evaluated by endoscopy with biopsy and cytology in follow-up examinations. When the ulcer caused by treatment entered the healing stage 4 weeks later, the patient was discharged. They were followed up by endoscopy 2, 3, and 6 months later, then every 6 months for 2 years, and thereafter once a year. To detect metastasis to the lungs, liver, and mediastinal lymph nodes, the patients were also followed up by chest X-ray, ultrasonic echography, and computed tomography. The curativity was graded two categories: cure or no cure. Although it is impossible to prove the cancer was cured by clinical examinations, we gave a definition of cure in this article when neither local sign of recurrence nor metastasis was proved by follow up examinations in one year. This was based on our experience in the treatment of superficial esophageal cancer. As PDT was indicated only for relatively early-stage cases, it always can reduce the cancerous tissue almost completely, even if it did not give a complete cure. On the other hand, if PDT failed to eradicate even a small portion of the cancerous lesion, the treatment was rendered useless. Therefore, a grade of partial response was classified as no cure in our evaluation (Mimura *et al.*, 1990).

### RESULTS

Of these eight superficial esophageal cancers, six were cured by initial PDT, whereas one lesion required another PDT to obtain a complete cure. The final rate of cure was 88% (7/8) as shown on Table 3.

### SIDE EFFECTS

The main toxic effects were photosensitivity and cutaneous reaction, seen in one (14.3%) of seven patients. They lasted several weeks. In laboratory tests, transient elevations of GOT and GPT were observed in another patient (14.3%, 1/7). They occurred in the second PDT and lasted 2 weeks. No other serious adverse reactions were seen.

### CASE

A 73-year-old woman with superficial esophageal cancer was treated by PDT with PHE and EDL. She could not undergo surgery, because of heart failure. The clinical course of PDT is shown by the endoscopic photographs in Figures 1–6. Endoscopically, the finding was suspicious for submucosal invasion and squamous cell carcinoma was diagnosed by biopsy. Figure 1 shows the finding before treatment, Figure 2 shows the finding after staining by iodine. The lesion was located in the middle part of the esophagus and spread over 7 cm$^2$. Figure 3 shows the finding immediately after the irradiation (60 J/cm$^2$) with the EDL, showing edematous swelling. One week later, a large, shallow ulcer developed, as shown in Figure 4. Four weeks later the ulcer healed as shown in Figure 5. Some

**Table 3** Rates of cure obtained by PDT with the EDL in superficial esophageal cancers, classified by depth

| *Depth of invasion* | *Number of cases* | *Number of cured cases (%)* |
|---|---|---|
| Mucosa | 4 | 4 (100%) |
| Submucosa | 4 | 3 (75%) |
| Total | 8 | 7 (88%) |

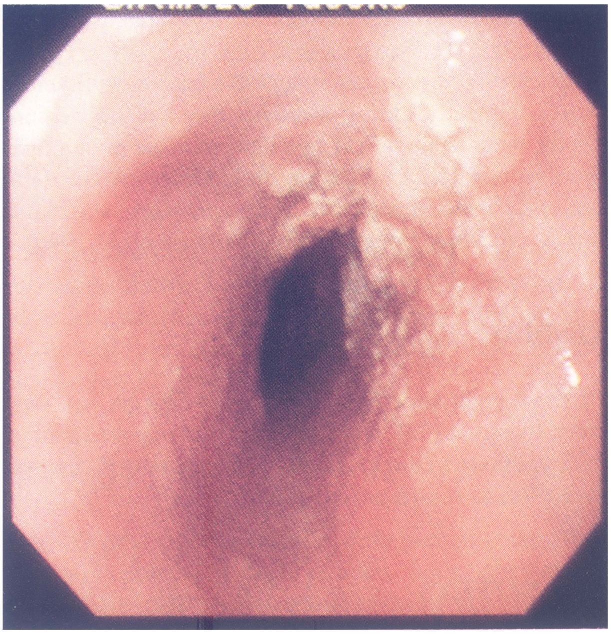

Figure 1 Before PDT treatment.

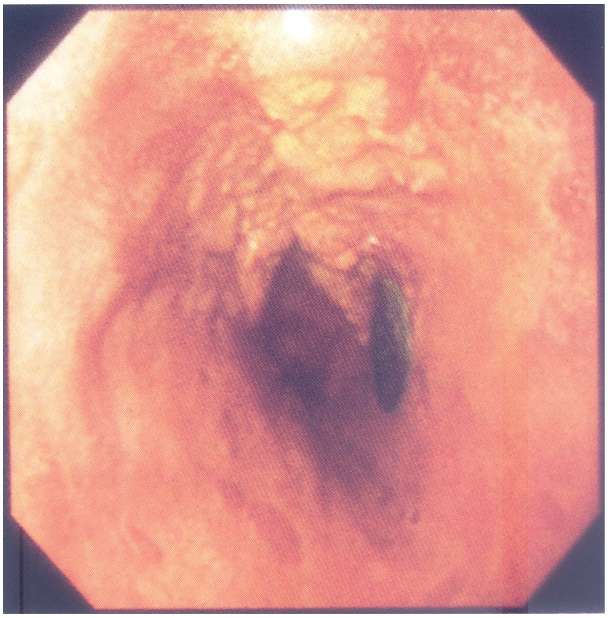

Figure 2 After staining with iodine. The $7cm^2$ lesion was located in the mid-thoracic esophagus and was endoscopically suspicious for submucosal invasion. Biopsy revealed squamous cell carcinoma.

minute nodules can be seen in the region of the scar; however, biopsy revealed no evidence of malignancy. Figure 6 shows the iodine stain finding 6 months later. Two small unstained spots can be seen in the scar lesion, but both were negative for malignancy histopathologically. Follow-up for 3 years after PDT demonstrated no recurrence of cancer.

### DISCUSSION

### Other Endoscopic Curative Treatments

To evaluate the role of PDT in the treatment of superficial esophageal cancer, other useful endoscopic curative methods should be taken into consideration. These methods can be divided into two types: local resection and local destruction. The former is intended to remove the lesion for histopathologic evaluation. A high frequency electric current is used to resect the lesion without causing bleeding. The indications of this method, initially developed by Momma and Makuuchi, were expanded by Inoue *et al.*, (1992). This method yields pathologic information such as depth of cancer invasion, histologic type, presence of lymph vessel invasion, and presence or absence of cancer at the margin of the specimen, and therefore can permit more exact analysis of prognosis in terms of curability and facilitate decisions concerning further surgical resection. However, applications of this method are limited by the nature of the cancerous lesion: that is, its size, depth of invasion, morphologic type, histologic type, and location. In general, the method is suitable for relatively small mucosal carcinomas.

Various techniques have been developed for the second type of treatment, local destruction, including microwave coagulation and Nd:YAG laser irradiation. Wider and deeper lesions can be destroyed using these techniques, but excessive treatment to obtain complete cure may cause perforation of the esophageal wall.

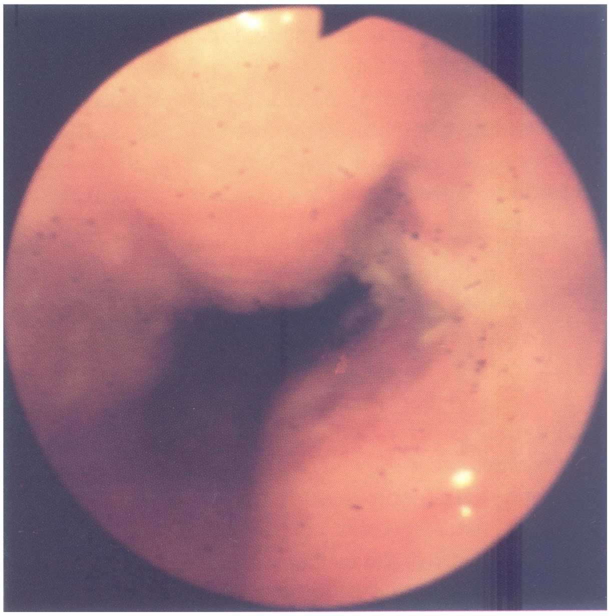

Figure 3 Immediately after 60 $J/cm^2$ PDT with the EDL showing edematous swelling.

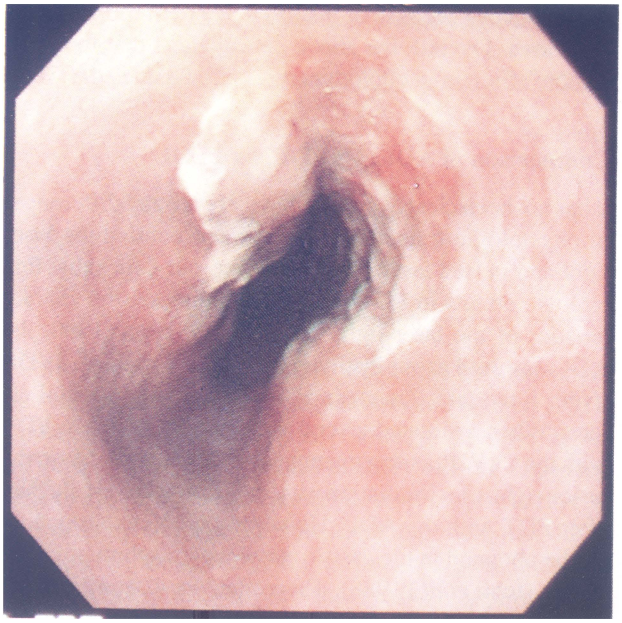

**Figure 4** One week later, a large, shallow ulcer had formed.

PDT is yet another approach to the local destruction of malignant tissue. Much progress in PDT was made during the first half of the 1980s, and good results have been reported on its use for the treatment of superficial esophageal cancers, especially depressed types, without any report of perforation (Okushima, 1987).

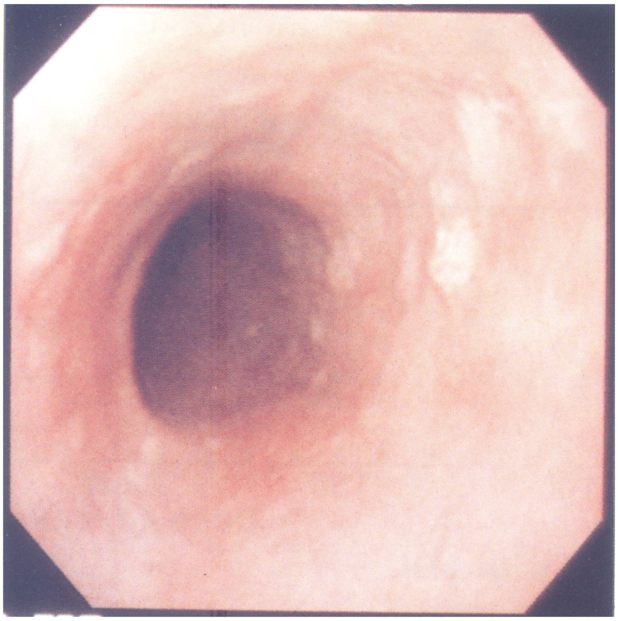

**Figure 5** Four weeks later, the ulcer had healed and some minute nodules could be seen in the region of the scar. However, biopsy showed no evidence of cancer.

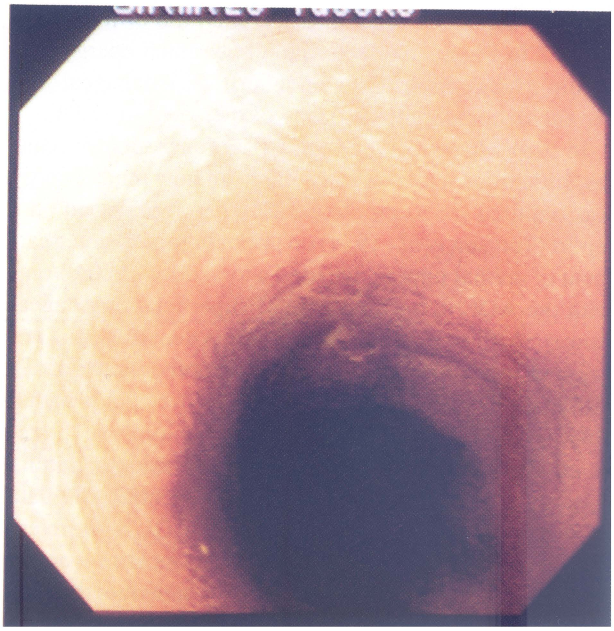

**Figure 6** Shows the iodine-stained feature at 6 months after PDT by electronic endoscope. Two small, unstained spots in the scar were both negative for malignancy by biopsy. This case has been followed up for 3 years after PDT and has shown no sign of recurrence.

Resection first should be considered to resect the entire lesion as much as possible, and PDT should be considered as the treatment of choice only for unresectable lesions.

### Side-Viewing Fiberscope

In PDT for gastric cancer, we used a side-view fiberscope (model GF-20, Olympus, Tokyo, Japan) to irradiate the lesion at an angle of 90°, keeping a certain distance between the fiber tip and the lesion since the first trial in 1981. However, in PDT for esophageal cancer, we used front-view fiberscope (model GIF-P10, GIF-XQ20) according to the traditional method of diagnostic endoscopy until 1991. Since the first trial using a side-view fiberscope in PDT for esophageal cancer in 1992 (Mimura and Imanishi, 1993), we have used a side-view fiberscope, model GF-20, in PDT for three cases with esophageal cancer, not only in PDT, but also in pretreatment examinations and follow-up examinations. In this procedure, patients with a lesion located on the right side can lie in a left lateral position as usual, whereas patients with a lesion located in the left side, especially from 7 to 10 o'clock, must lie in a right lateral position. This enabled photoradiation of esophageal cancer from a 90° angle without any difficulty.

### Dose and Response

The relationship between response (cure or no cure) and irradiated energy intensity (dose: $J/cm^2$) was evaluated in terms of depth of cancer invasion and kind of laser used in PDT in Figure 7 to determine how much energy was required for a complete cure in superficial esophageal cancer and to compare the efficacy of the ADL and EDL.

In 1981 when we started using PDT for esophageal cancer, little information was available on how much energy was required for a complete cure. Therefore, based on a report (Tomson *et al.*, 1974) on laser photoradiation treatment of animal tumors, we used a total dose of 204 J/cm$^2$ with the ADL in our first case of superficial esophageal cancer. Although, such high-energy densities did not cause any side effects such as perforation or acute mediastinitis, it became clearer by our later investigations that although a high dose did not necessarily improve the response, uniform irradiation of the entire lesion including a marginal zone of intact mucosa was essential. Our data in Figure 7 show that 90 J/cm$^2$ with the ADL is sufficient for treatment of mucosal esophageal cancer. However, the ADL laser beam could not sufficiently penetrate the submucosal layer. With EDL, 60 J/cm$^2$ is sufficient not only for mucosal cancer but also for submucosal cancer. In the one case in which we could not obtain a complete cure with the EDL, a small shallow cancer nest was left behind in the mucosa because of insufficient extent of the irradiation area. Overall, the above data suggest that the EDL is more efficient in photodynamic action than is the ADL.

### Comparison of ADL, CuVDL, and EDL

It has not been clearly proved that there are any differences in physiologic transmissivity between continuous laser light and pulsed laser light. However, our data show that biologic effects in PDT varied depending on the characteristics of the laser light. Between 1981 and 1990, the ADL was used in our institute. In 1988, we tried a CuVDL (Niic, Kawasaki, Japan) in PDT, anticipating special effects. When we treated several early gastric cancers by PDT with the CuVDL, subsequent hemorrhagic necrosis on the lesion right after irradiation was found more obviously than after PDT with the ADL. However, complete cure could not be obtained in the gastric cancers with submucosal invasion (Ichii *et al.*, 1988). Thereafter, we discontinued its use, because we could not find any advantages of the CuVDL in clinical use in early gastric cancer.

We first used the EDL for PDT, in 1990, at 4 mJ per pulse, 400 kW of peak power, and 40 pulses per second

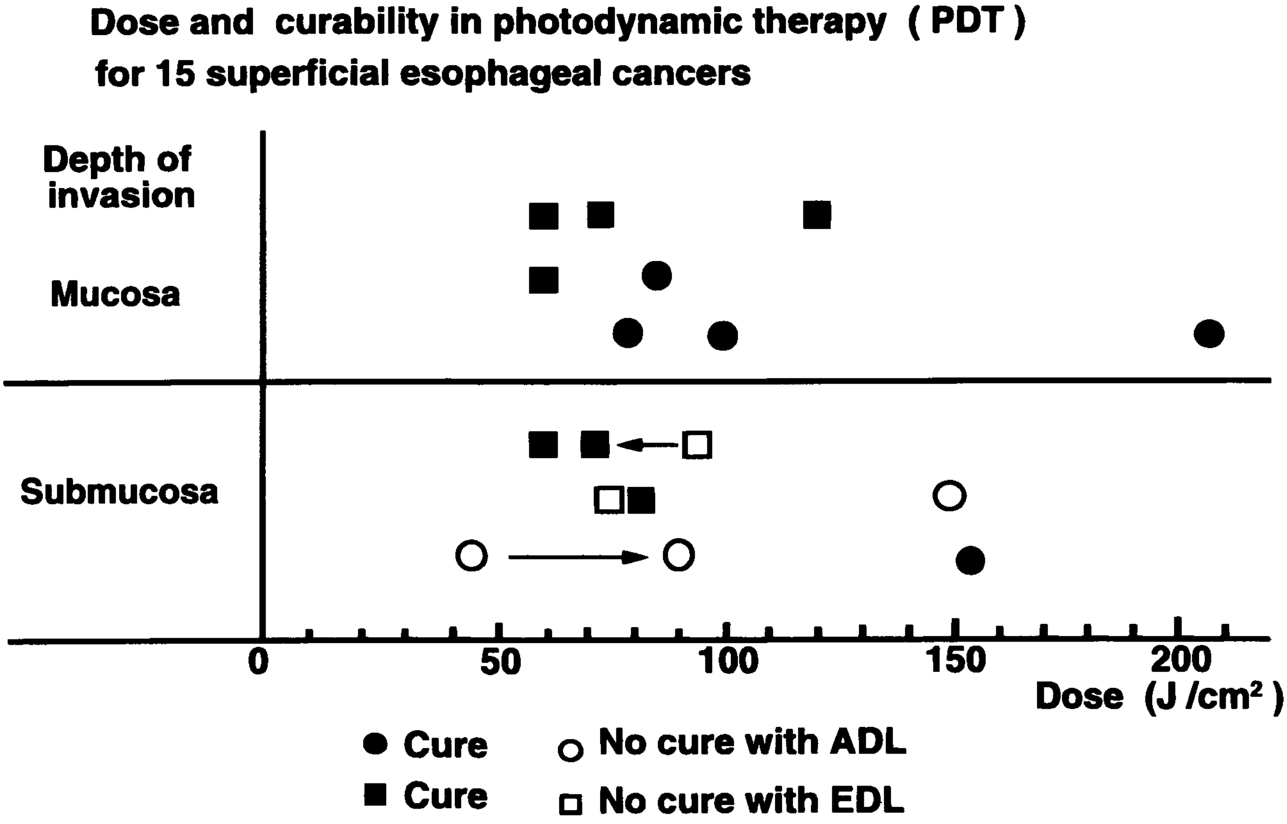

**Figure 7** The relationship between response (cure or no cure) and photoradiated energy intensity (dose: J/cm$^2$) in terms of depth of cancer invasion and kind of laser used in PDT. Arrows indicate the same case undergoing a second PDT.

Table 4 Laser characteristics

| *Type of laser* | *Pulse energy* | *Peak power* | *Pulse width* | *Pulse frequency* | *Average output* |
|---|---|---|---|---|---|
| Argon dye laser | ——— | (300 mW) | ——— | ——— | 300 mW |
| Copper vapor dye laser | 0.055 mJ | 2.5 kW | 22 nsec | 5,500 Hz | 300 mW |
| Excimer dye laser | 4 mJ | 400 kW | 10 nsec | 40 Hz | 160 mW |

for the allowable maximum, to protect the 400-μm single quartz fiber from destruction (Mimura and Okuda, 1991). These characteristics of the three kinds of laser are shown in Table 4. With the EDL, the efficacy of PDT improved not only for esophageal cancer but also for gastric cancer. It is difficult to determine the most important individual factor determining the biologic effect of a pulsed laser in PDT, but our studies suggest that the most important factor is peak power. We postulate that pulsed laser light has several functions, not only of activation of HpD and heat, but also an unknown effect. It may be the effect of a shock wave (Mimura *et al.*, 1992).

### CONCLUSION

PDT with PHE and the EDL is a safe and promising alternative for the treatment of patients with superficial esophageal cancer who are poor risks for surgery and in whom mucosal resection is difficult.

### ACKNOWLEDGMENTS

This work was supported in part by a Grant-in-Aid for Cancer Research (5–38) from the Ministry of Health and Welfare of Japan.

### REFERENCES

Dougherty T. J., Lawrence G., Kaufman J. H. *et al.* (1979) Photoradiation in the treatment of recurrent breast carcinoma. *JNCI*, 62:231–237.

Fuji Film (1993) Fuji film optical filter manual, (in Japanese).

Hayata Y., Kato H., Konaka C. *et al.* (1982) Hematoporphyrin derivative and laser photoradiation in the treatment of lung cancer. *Chest*, 81:269–277.

Hirano T., Suzuki K., Ishizuka M. *et al.* (1990) Pulse Laser Application for PDT. Proceedings of the 11th Annual Meeting of the Japanese Society for Laser Medicine, 103–106 (in Japanese).

Ichii M., Mimura S., Tatsuta M. *et al.* (1988) Photodynamic therapy of early gastric cancer with a copper vapor-dye laser. *J Jpn Soc Laser Med*, 9:125–126 (in Japanese).

Inoue H., Endo M., Takeshita K. *et al.* (1992) A new simplified technique of endoscopic esophageal mucosal resection using a cap-fitted panendoscope (EMRC). *Surg Endosc*, 6:264–265.

Kato H., Horai T., Furuse K. *et al.* (1993) Photodynamic therapy for cancers: a clinical trial of porfimer sodium in Japan. *Jpn J Cancer Res*, 1209–1214.

Lipson R. L., Baldes E. J. (1960) The photodynamic properties of a particular hematoporphyrin derivative. *Arch Dermatol*, 82:508–516.

Mimura S., Ichii M., Imanishi K. *et al.* (1990) Evaluation of photodynamic therapy. *Dig Endosc*, 2:265–274.

Mimura S., Ichii M., Okuda S. (1993) Further investigation in photodynamic therapy. *J Jpn Soc Laser Med*, 14:45–50 (in Japanese).

Mimura S., Ichii M., Okuda S. (1992) Photodynamic therapy for early gastric cancer using excimer dye laser. In: Spinelli P., Fante M.D., Marchesini R. (eds.): *Photodynamic Therapy and Biomedical Lasers*. Elsevier Science Publishers B.V., Amsterdam.

Mimura S., Ichii M., Okuda S. (1991) Present status of photodynamic therapy for esophageal cancer and gastric cancer. *Biotherapy*, 552–559 (in Japanese).

Mimura S., Imanishi K. (1993) Irradiation technique in photodynamic therapy for esophageal cancer using side-viewing fiberscope. Proceedings of 44th JGES, *Gastroenterol Endosc*, 35:1449 (in Japanese)

Mimura S., Okuda S. (1993) Photodynamic therapy for esophageal cancer and gastric cancer. *JJCDO*, 49–55 (in Japanese).

Mimura S., Okuda S. (1991) Photodynamic Therapy with excimer dye laser for superficial esophageal cancer and early gastric cancer. Proceedings of the 12th Annual Meeting of the Japanese Society of Medicine, 107–110 (in Japanese).

Okuda S., Mimura S., Otani T. *et al.* (1984) Diagnostic laser fluorescence and photochemotherapy. In: Montorsi W - Veronesi (ed.) *I Tumori Gastroenterici*. Milano.

Okunaka T., Kato H., Konaka C. (1992) A comparison between argon-dye and excimer-dye laser for photodynamic effect in transplanted mouse tumor. *Jpn J Cancer Res*, 83:226–231.

Okushima N. (1987) Endoscopic photodynamic therapy for esophageal cancer. *Gastroenterol Endosc*, 29:1105–1115 (in Japanese).

Star W. M., Marijnissen J. P. A., Van den Berg-Blok A. E. *et al.* (1986) Destruction of rat mammary tumor and normal tissue microcirculation by hematoporphyrin derivative photoradiation, observed in vivo in sandwich observation chambers. *Cancer Res*, 46:2532–2540.

Tomson S. H., Emmett E. A., Fox S. H. (1974) Photodestruction of mouse epithelial tumors after oral acridine orange and argon laser. *Cancer Res*, 34:3124–3127.

Weishaupt K. R., Gomer C. J., Dougherty T. J. (1976) Identification of singlet oxygen as the cytotoxic agent in photodynamic inactivation of a murine tumor. *Cancer Res*, 36:2326–2329.

